# An Investigation of the Effects of Riboflavin Concentration on the Efficacy of Corneal Cross-Linking Using an Enzymatic Resistance Model in Porcine Corneas

**DOI:** 10.1167/iovs.17-22994

**Published:** 2019-02

**Authors:** Naomi A. L. O'Brart, David P. S. O'Brart, Nada H. Aldahlawi, Sally Hayes, Keith M. Meek

**Affiliations:** 1 Moorfields Eye Hospital, City Road, London, United Kingdom; 2 Keratoconus Research Institute, Department of Ophthalmology, St. Thomas' Hospital, London, United Kingdom; 3 Structural Biophysics Research Group, School of Optometry and Vision Sciences, Cardiff University, Maindy Road, Cardiff

**Keywords:** keratoconus, corneal cross-linking, riboflavin, enzymatic digestion

## Abstract

**Purpose:**

To investigate riboflavin concentration on enzymatic resistance following corneal cross-linking (CXL).

**Methods:**

Ninety-six porcine eyes were divided into five groups in two treatment runs. Group 1 remained untreated. Group 2 received riboflavin 0.05%, group 3 riboflavin 0.1%, group 4 riboflavin 0.2%, and group 5 riboflavin 0.3%. Treated eyes underwent CXL with ultraviolet A at 9 mW/cm^2^ for 10 minutes. Eight-millimeter discs from each cornea were submerged in pepsin digest solution. In the first run, disc diameters were measured daily. After 10 days, dry weights were recorded from five samples in each group. In the second run, dry weights were recorded in five samples in each group at 10 and 20 days.

**Results:**

CXL-treated corneas took longer to digest than untreated (*P* < 0.001). Although eyes treated with higher riboflavin concentrations generally took longer to digest, there were no significant differences between groups (*P* = 0.3). Dry weights at 10 days demonstrated, with each increase in concentration, an increase in weight of residual undigested tissue (*P* < 0.001). In the second run, with each increase in riboflavin concentration there was an increase in weight of residual tissue (*P* < 0.001) at 10 days. At 20 days, the dry weight was lower with 0.05% riboflavin compared to 0.3% (*P* < 0.001) and 0.2% and 0.1% solutions (*P* < 0.05), with no other difference between groups.

**Conclusions:**

There is a consistent dose-response curve with higher concentrations of riboflavin achieving greater CXL efficacy, suggesting that manipulation of riboflavin dosage as well as the UVA protocol can be used to optimize CXL.

The current management of corneal ectatic diseases such as keratoconus depends on the severity and extent of the degree of irregular astigmatism.^1^ Mild cases can be corrected with spectacles and soft toric contact lenses.^2^ However, such modalities are limited in their effectiveness as the cornea becomes more irregular. In advanced disease, special soft, rigid gas-permeable, and scleral contact lenses become a more suitable solution to restore vision.^3^ Despite the use of such special contact lenses, studies indicate that over 25% of patients with keratoconus can progress to such an extent that they require corneal transplantation,^4^ with this disease remaining the most common indication for penetrating keratoplasty in Europe, Australia, South America, Africa, and the Middle East.^5^

The introduction of cross-linking (CXL) has heralded a new era in the treatment of corneal ectatic disorders. CXL utilizing riboflavin/ultraviolet A (UVA) is now a widely established intervention that has been shown to halt the progression of the disease process in keratoconus, post-LASIK ectasia, and pellucid marginal degeneration.^6^,^7^ The rationale of CXL is to enhance the biomechanical rigidity of the cornea^6^,^7^ and its resistance to enzymatic digestion^8^ by creating cross-links between both collagen and proteoglycan molecules within the corneal stroma.^9^–^11^ Not only does CXL appear to stabilize corneal ectasia at a stage when contact lenses can still be utilized, it also has been shown in many treated individuals to reduce topographic steepness and corneal high-order aberrations, resulting in improved vision.^12^ Indeed, a recent study in the Netherlands demonstrated a significant reduction in the number of corneal transplants for keratoconus following the nationwide introduction of CXL.^13^

The standard CXL (SCXL) protocol, first described clinically by Wollensak et al.^7^ in 2003, involves debridement of the central 9 mm of corneal epithelium, followed by soaking the exposed stromal surface with 0.1% riboflavin for 30 minutes and irradiation with 370-nm UVA light with an intensity of 3.0 mW/cm^2^ for 30 minutes (a total dose of 5.4 J/cm^2^). This protocol requires more than 1 hour of treatment time. To shorten this procedure time, given the large potential numbers of patients with progressive keratoconus requiring CXL and its resultant burden to health service delivery, and to improve patient and surgeon convenience alike, accelerated CXL (ACXL) protocols have been introduced.^14^ These ACXL techniques are based on the Bunsen-Roscoe law of photochemical reciprocity and are modeled on the understanding that the same photochemical effect can be achieved with a reduced irradiation interval, provided that the total energy level is kept constant by a corresponding increase in irradiation intensity.^15^ However, as has been demonstrated with other photochemical reactions, this law may be valid only within a certain dose range and varies with different types of photochemical processes.^16^ Certainly, while efficacy has been demonstrated, it appears both in the laboratory^17^ and clinically^18^,^19^ that ACXL may be less effective than SCXL, although the minimum effective amount of CXL needed for stabilization of ectasia has not yet been established.^17^,^20^

The precise reasons for the reduced efficacy of ACXL protocols are unclear, and the exact mode of action of CXL at a molecular level is undetermined.^21^ It is postulated that riboflavin acts as a photosensitizer to produce both oxygen singlets and riboflavin triplets,^22^ which then drive the CXL process within the corneal stroma.^23^ What is understood is that oxygen is essential to drive the process, and in the absence of oxygen, CXL is impaired.^24^ It has been hypothesized that the reduced efficacy associated with ACXL protocols might be the result of more rapid oxygen depletion.^25^,^26^ Kameav et al.^27^ showed that, oxygen consumption occurs within seconds during UVA irradiation and that following cessation of irradiation, oxygen levels can take several minutes to be restored. As such it has been hypothesized that by pulsing the UVA light during irradiation, oxygen levels within the stroma might be replenished/maintained so that the CXL process is not compromised.^28^,^29^ Other investigators have advocated that in addition to pulsing optimization of ACXL protocols can be achieved by extending the UVA dosage by 30% to 40%.^30^,^31^ Indeed, in a laboratory study utilizing the same resistance to enzymatic digestion methodology employed in this present study, our group demonstrated increased efficacy with both extended and pulsed UVA dosages with ACXL protocols, although the results suggested that the distribution of cross-links may be different compared to SCXL.^23^ As described above, to date virtually all research directed at optimizing CXL protocols has been targeted at manipulating the UVA irradiation dosage of the treatment. Indeed, except for some transepithelial CXL protocols,^32^ published CXL research, including those with varying riboflavin formulations, has almost universally maintained a riboflavin concentration of 0.1%. Almost 15 years after publication of the first clinical paper,^7^ the optimal stromal riboflavin dosage for CXL is yet to be determined.^22^ This present study aims to investigate this issue by determining the efficacy of CXL using an enzymatic (pepsin) resistance model in ex vivo porcine corneas with varying concentrations of riboflavin (0.05%, 0.1%, 0.2%, and 0.3%).

## Methods

A total of 96 fresh porcine eyes were utilized in this study. All had transparent corneas and an intact corneal epithelium on inspection. They were obtained and transported on ice from a local European Community licensed abattoir and used within 12 hours of death. The experiment was conducted using two separate treatment runs to ensure the consistency of the results. In the first run, 50 eyes were utilized, and in the second, conducted 8 weeks later, 46 eyes were used.

Our pepsin digestion methodology has been published previously.^17^,^23^ We chose this method as it facilitates relatively slow digestion rates. This has allowed us in our past studies to not only detect significant differences in the digestion rates in terms of disc diameter measurements and time to complete digestion but also to measure dry weights at 10 days, which has highlighted significant differences between treatment protocols that are not evident by just measuring disc diameters and time to digestion alone.^17^,^23^ In addition, using this same protocol we have repeatedly shown that there are no differences in digestion times in nonirradiated corneas that have not received riboflavin drops and those soaked in riboflavin for 30 minutes.^17^,^23^ This allowed us in this study to reduce the number of control groups and just use de-epithelialized, nontreated, nonirradiated corneas as a sole control group. In brief, following complete debridement of the corneal epithelium using a single-edged razor blade, the eyes were divided randomly into the five groups described in Table 1, in which group 1 (no riboflavin administered and no UVA exposure) served as an untreated control and groups 2, 3, 4, and 5 received varying concentrations of riboflavin solution (0.05%, 0.1%, 0.2%, 0.3%, respectively) for a period of 30 minutes. All riboflavin solutions contained 20% dextran and were identical in formulation except for the riboflavin concentration. The riboflavin was applied using a 10-mm suction ring/container (J2294; E. Janach srl, Como, Italy) that was completely filled to its brim with the relevant riboflavin solution concentration after being placed and suctioned over the central cornea. Following the 30-minute riboflavin diffusion period, all eyes in groups 2, 3, 4, and 5 underwent irradiation using a CCL-VARIO corneal cross-linking UVA lamp (Peschke Meditrade GmbH, Huenenberg, Switzerland) with a wavelength of 365 nm and a 9.0-mm aperture, with an intensity of 9 mW/cm^2^ for 10 minutes (total energy dose 5.4 J/cm^2^). An accelerated treatment protocol was chosen, as we needed to treat 44 eyes in each experimental run and wished to use only one UVA irradiation device so that fluence levels and UV beam profile were consistent between treatments. If we had used the standard protocol of 3 mW/cm^2^ for 30 minutes, it would have taken 22 hours to complete the treatments in radiation time alone. This would mean that the eyes being treated last would be well over 24 hours old since time from enucleation. Even though we treated one eye from each group sequentially to minimize any effects of some eyes being treated later than others, we did not want to include any eyes in which treatment occurred after 24 hours. This was undertaken to diminish any effects of natural decomposition, endothelial and epithelial cell death with subsequent changes in corneal hydration, and possible fungal/bacterial contamination. By using an accelerated treatment of 9 mW/cm^2^ for 10 minutes, we could reduce the total irradiation time to just over 7 hours and treat all eyes with 12 hours.

**Table 1:** Treatment Groups for the Two Separate Experimental Runs, Detailing the Differing Riboflavin Concentrations Utilized

**Groups**	**Riboflavin Soak Time**	**CXL Time**	**No. of Eyes**	
			**Run 1**	**Run 2**
1. Untreated corneas	30 minutes	9 mw/cm^2^ for 10 min	6	6
2. Riboflavin 0.05%	30 minutes	9 mw/cm^2^ for 10 min	11	10
3. Riboflavin 0.1%	30 minutes	9 mw/cm^2^ for 10 min	11	10
4. Riboflavin 0.2%	30 minutes	9 mw/cm^2^ for 10 min	11	10
5. Riboflavin 0.3%	30 minutes	9 mw/cm^2^ for 10 min	11	10

Central corneal thickness measurements were taken manually using a handheld ultrasonic pachymeter (Pachmate 55; DGH Technology, Inc., Exton, PA, USA) after epithelial removal, after riboflavin administration, and after UVA irradiation in the treatment groups 2, 3, 4, and 5 in the first experimental run (44 eyes).

On completion of the treatments, an 8-mm full-tissue-thickness biopsy was trephined from the center of each cornea. The corneal discs were placed in individual sealed tubes containing 5 mL pepsin digest solution, 1 g ≥ 500 U/mg pepsin from porcine gastric mucosa (Sigma-Aldrich Corp., Dorset, UK) in 10 mL 0.1 M hydrochloric acid at pH 1.2 and incubated them in a water bath at 23°C. As previous studies have indicated that CXL results in cross-links not only at the collagen fibril surface but also in the proteoglycan network surrounding the collagen,^11^ we selected pepsin for enzymatic digestion as it is a nonspecific endopeptidase that breaks down both collagen and proteoglycan core proteins.

To reduce the risk of fungal/bacterial contamination, sterility was maintained by undertaking the preparation of the sample specimens with sterile instruments, which were cleaned with an ethanol alcohol spray (Spiriclens DEB; Ecolab Ltd., Leeds, UK) between specimen preparation. The sealed tubes into which the corneal discs were placed were clean and sterile prior to usage. To avoid contamination, from the water bath, 30 mL chlorine-based disinfecting solution (Milton sterilizing fluid; Milton Pharmaceutical Company, Bournemouth, UK) was added to the bath water every 3 days and the water replaced every 12 days. All instruments used when handling samples for measurement were cleaned before and between sample measurement and left to dry using ethanol alcohol spray. At day 26, in the first experimental run the pepsin digest solution in each sealed tube was replaced, both to enhance further digestion and to attempt to maintain sterility.

### First Experimental Run

In the first experimental run (involving 50 eyes), electronic digital calipers were used to perform daily measurements of the longest diameter of the anterior surface of each corneal disk. This was facilitated by microscopic examination at 10× magnification after the disk had been poured into a sterile petri dish and separated from the pepsin digest solution using a sterilized pipette. This magnified examination allowed the longest diameter of the disc to be identified and precisely measured with the electronic calipers. Measurements were performed until the tissue could no longer be distinguished from the surrounding pepsin solution, at which time point the tissue was considered to have undergone complete digestion.

Measurements of anterior corneal disc diameter, rather than stromal thickness, were used to assess the rate of enzymatic digestion as the corneal discs underwent significant posterior stromal swelling (in the vertical direction) during immersion in pepsin digest solution, and the more-resistant anterior portion of the cornea then separated from the posterior stroma after 4 to 6 days.^17^,^23^

To further evaluate the effect of different riboflavin concentrations on corneal enzymatic resistance, five randomly selected corneal discs from each CXL-treated group (groups 2, 3, 4, and 5) were removed from the pepsin digest solution after 10 days, placed in a 60°C oven, and weighed daily until a constant dry weight was obtained. The average dry weight was then calculated for each group. There were no differences between the groups in the mean times each of the groups were in the oven (median 7 days). This methodology was undertaken to ensure the samples were completely dehydrated and the true dry tissue weight verified.

The remaining six discs in each of the CXL treatment groups 2, 3, 4, and 5 remained in pepsin digest solution and were measured daily, as described above, until digestion was complete. At day 26 it appeared that the discs had stopped digesting so the digest solution in each sealed tube was replaced with freshly prepared pepsin digest solution (with the same formulation as described previously), and the temperature of the water bath was increased to 26°C to accelerate the digestion process.

### Second Experimental Run

To ensure the consistency of the results, a second experimental run of 50 eyes was conducted (Table 1). While previous enzymatic digestion studies from our group^17^,^23^ have indicated that the corneal disc diameter measurements appear to provide information about the structural integrity of the most anterior layers of the cornea, they can be subject to variation. This occurs especially in the later stages of digestion when the tissue tends to lose its circular shape and become irregular in outline, making the longest diameter of the tissue difficult to determine. As the dry weight measurements represent the total mass of undigested tissue and negate any of the problems associated with tissue shape and between-sample differences in hydration, in the second experimental run we replaced reliance on this more-reproducible methodology. In this run, after being immersed in pepsin solution, the discs remained in their sealed containers and were incubated at 23°C until day 10, when five corneal discs from each CXL treatment group (groups 2, 3, 4, and 5) were removed from the digest solution and placed in the 60°C oven until a constant dry weight was obtained and the average corneal dry weight calculated for each group. This process was repeated at 20 days, when the remaining five corneal discs from each group were removed from the pepsin digest, placed in the 60°C oven, and their constant dry weights recorded. The digest solution was not renewed during this second run, and the water bath temperature was maintained at 23°C.

### Statistical Analysis

Data are shown as average measurements (± SD) for corneal thickness, dry weight, and complete digestion time. Measurements of corneal disk diameter are presented as a daily cumulative measurement for each treatment group. Statistical analysis was performed using a 1-way ANOVA and Bonferroni multiple comparisons in a depth-wise manner. All statistical analyses were performed with the Statistical Package for the Social Sciences (SPSS Statistics 20; IBM, Armonk, NY, USA). A probability value of *P* < 0.05 was considered significant.

## Results

### Corneal Thickness

**Figure 1:**
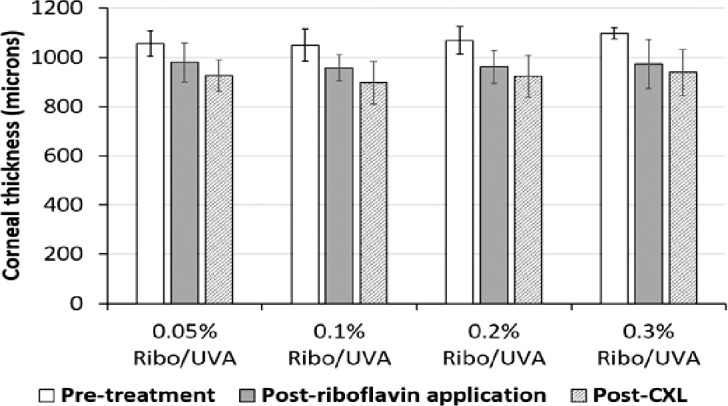
Corneal thickness measurements for each group in the first experimental run are shown before treatment (after epithelial debridement), following riboflavin administration for 30 minutes, and after CXL (n = 11 per group). Data are shown as the mean value ± SD.

The average stromal thicknesses pretreatment (after epithelial debridement), after riboflavin administration and after UVA irradiation in the first experimental run are shown in Figure 1. There was a significant decrease in thickness following CXL (from pretreatment to after CXL) in all groups (*P* < 0.001), but there were no differences in thicknesses between the groups at any stage.

### Time Taken for Complete Digestion

**Figure 2:**
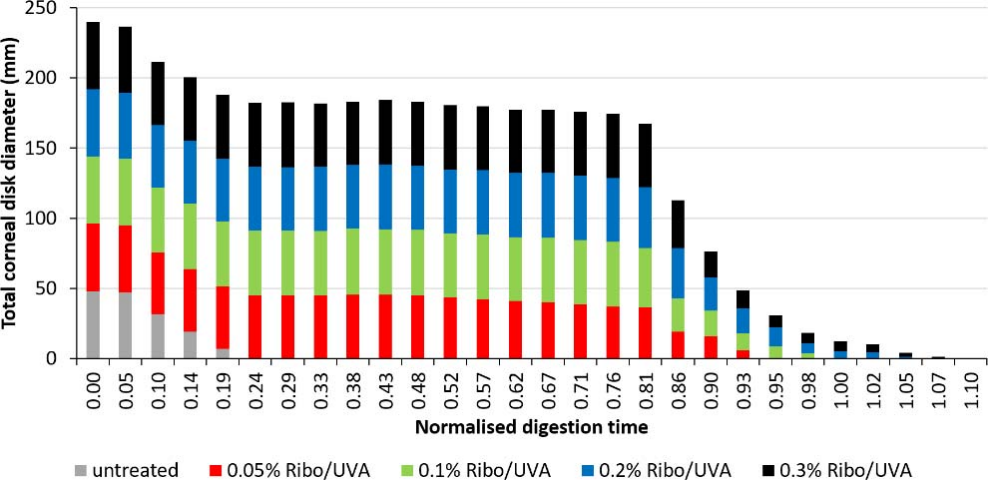
The summed longest diameter of the corneal discs in each group in the first experimental run is shown as a function of time, which has been normalized against the maximum time taken for corneas cross-linked using the standard 0.1% riboflavin formulation (42 days) to undergo complete digestion. The discs appeared to stop digesting at approximately day 26 (normalized time of 0.62) so temperature was increased to 26°C and the digest solution renewed.

As previously reported,^17^,^23^ in all corneal discs significant stromal swelling, in a posterior–anterior direction, was documented within 24 hours of submersion in the pepsin digest solution. By 4 to 6 days, the anterior portion of each corneal disc had separated from the posterior portion, and by days 8 to 10 the posterior portion of each disc had undergone complete digestion. The anterior portion of the corneal discs persisted to allow daily measurements of diameter (Fig. 2; Table 2).

**Table 2:** Time in Days Taken for Complete Digest to Occur in All Groups

**Group**	**Time Taken for Complete Digestion to Occur**		
	**Minimum, d**	**Maximum, d**	**Average, d (±SD)**
Untreated	6	10	8 (±2)
0.05% Ribo/UVA	36	40	39 (±2)
0.1% Ribo/UVA	36	42	39 (±2)
0.2% Ribo/UVA	39	45	41 (±2)
0.3% Ribo/UVA	38	46	41 (±3)

Ribo/UVA, Riboflavin/UVA.

The time required for complete digestion of the CXL-treated corneal discs (groups 2, 3, 4, and 5) was significantly longer than that required for the untreated specimens in group 1 (*P* < 0.001) (Fig. 2; Table 2). After 10 days, all untreated corneas (group 1) had undergone complete digestion in both experimental runs. Based on cumulative measurements of corneal disc diameter for each treatment group, the irradiated corneas treated with a higher concentration of riboflavin appeared to take longer to undergo complete digestion than those treated with lower concentrations of riboflavin. However, this was largely due to the persistence of a minority of samples within the higher riboflavin concentration groups (Fig. 2), and the average time taken for complete digestion to occur did not differ significantly between the CXL-treated groups (*P* = 0.3) (Table 2).

### Dry Weight Measurements

After 10 days in the pepsin digest solution, only the CXL-treated corneas remained, with all the untreated discs being completely digested in both experimental runs (Fig. 2; Table 2).

### First Experimental Run

**Figure 3:**
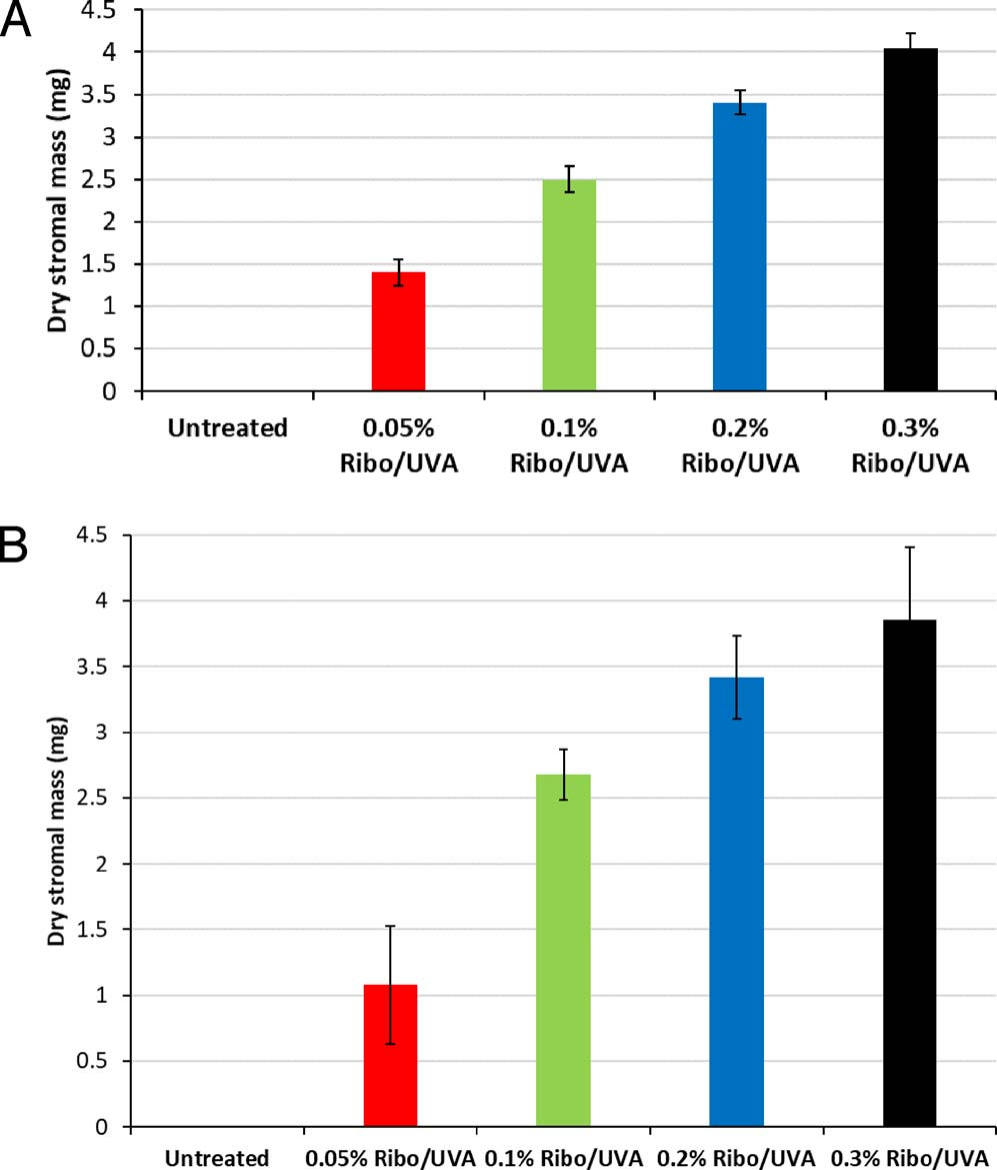
(A) Average stromal dry weight of the untreated and treated groups (n = 5 samples per group) at day 10 in the first experimental run. Error Bars: ±1 SD. There was a statistically significant difference in dry weights between all groups (P < 0.001) at 10 days. (B) Average stromal dry weight of the untreated and CXL-treated groups (n = 5 samples per group) at day 10 in the second experimental run. Error Bars: ±1 SD. There was a statistically significant difference in dry weights between all groups (P < 0.001) at 10 days.

In the first experimental run, there was a statistically significant difference in dry weight measurements between all treated groups (2, 3, 4, and 5) at 10 days (*P* < 0.001). For each group with an increased riboflavin concentration, there was a significant increase in the dry weight of residual undigested corneal tissue, denoting an increased resistance of the tissue to pepsin digestion (*P* < 0.001) (Fig. 3A; Table 3).

**Table 3:** Average Stromal Dry Weight of Control Group 1 and Treated Groups 2, 3, 4, and 5 (n = 5) at Day 10 in First and Second Experimental Runs

**Group**	**Mean Dry Weight After 10 Days of Digestion, mg ±1 SD**	
	**Run 1**	**Run 2**
Untreated	0	0
0.05% Ribo/UVA	1.4 ± 0.16	1.08 ± 0.45
0.1% Ribo/UVA	2.5 ± 0.16	2.68 ± 0.19
0.2% Ribo/UVA	3.4 ± 0.14	3.42 ± 0.32
0.3% Ribo/UVA	4.04 ± 0.18	3.86 ± 0.54

### Second Experimental Run

Similarly, in the second experimental run, there was a statistically significant difference in dry weight measurements between all treated groups (groups 2, 3, 4, and 5) at 10 days (*P* < 0.001). For each group with an increased riboflavin concentration, there was a significant increase in the dry weight of residual undigested corneal tissue, denoting an increased resistance of the tissue to pepsin digestion (*P* < 0.001) (Fig. 3B; Table 3), with comparable average dry weights for each of the treatment groups in the two experimental runs (Table 3).

**Figure 4:**
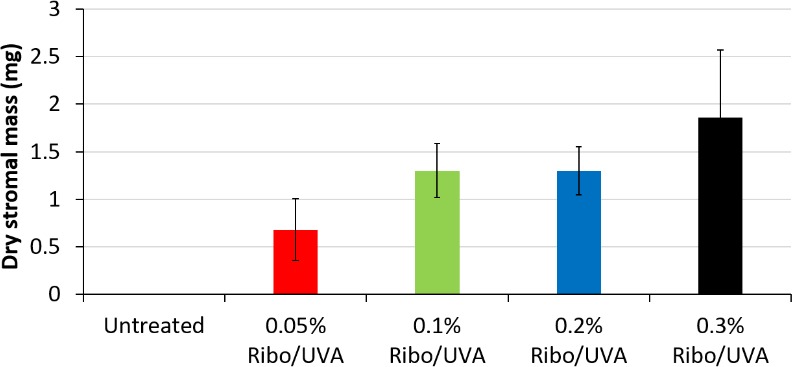
Average stromal dry weight (milligrams) of control group 1 and treated groups 2, 3, 4, and 5 (n = 5) at day 20 in a second experimental run. Error Bars: ±1 SD. The average stromal dry weight was significantly lower in the 0.05% riboflavin treatment group (group 2) than in the 0.3% (P < 0.001) and the 0.2% and 0.1% treatment groups (P < 0.05) (groups 3, 4, and 5).

In the second experimental run at 20 days, measurement of the average stromal dry weight was significantly lower in the 0.05% riboflavin treatment group (group 2) than in the 0.3% (*P* < 0.001) and the 0.2% and 0.1% treatment groups (*P* < 0.05) (groups 3, 4, and 5). There was no significant difference between 0.1%, 0.2%, and 0.3% riboflavin/UVA treated groups (groups 3, 4, and 5) at 20 days of digestion (Fig. 4; Table 4).

**Table 4:** Average Stromal Dry Weight of the Untreated and CXL-Treated Groups (n = 5 samples per group) at Day 20 in the Second Experimental Run

**Group**	**Mean Dry Weight at 20 Days in Second Experimental Run, mg ±1 SD**
Untreated	0
0.05% Ribo/UVA	0.68 ± 0.33
0.1% Ribo/UVA	1.3 ± 0.28
0.2% Ribo/UVA	1.3 ± 0.25
0.3% Ribo/UVA	1.86 ± 0.71

## Discussion

SCXL has been shown to be efficient in arresting the progression of corneal ectatic disease in most treated eyes with up to 10 years follow-up.^33^,^34^ Although numerous modifications of the epithelium-off CXL technique have been postulated, these have almost universally focused on variation in the UVA protocol and additives to the standard riboflavin 0.1% solution.^21^ Indeed, except for some epithelium-on CXL formulations that utilize 0.25% riboflavin concentrations,^32^ virtually all currently used epithelium-off protocols use a riboflavin concentration of 0.1%.^21^,^22^ Despite the passage of almost two decades since the first human clinical CXL treatments,^7^ the optimum riboflavin dosage for CXL is undetermined,^22^ but to fully evaluate the efficacy and safety of any drug, it is vital to determine its dose-response curve.^35^

There are multiple problems with determining this dose-response curve, the not least of which is the difficulty in measuring the biomechanical changes induced by CXL. As yet there are no reliable ways to measure these changes in vivo,^36^ a problem that compounded by the fact that any measurable clinical changes take months to become evident and years to stabilize.^33^,^34^ Even in vitro, the measurement of biomechanical changes after CXL are beset with difficulty. Standard biomechanical measurement methodologies, such as stress-strain extensiometry, have inherent deficiencies when applied to the assessment of biological tissues such as the cornea.^37^ Such biological tissues do not behave like metals and polymers with homogeneous chemical/molecular bonds but rather have nonhomogeneous chemical bonds and molecular interactions that result in viscoelastic material properties. In addition, strip specimens from corneas are originally part of a spherical surface, so the length of the strip along its longitudinal centerline is longer than its sides and there is variation in thickness between the corneal center and its periphery.^37^ All these factors can lead to poor measurement reliability, which has led investigators to explore other potential methodologies such as inflation techniques,^38^ scanning acoustic microscopy,^39^ and Brillouin microscopy,^40^ none of which have yet to become a reliable gold standard measurement technique to determine biomechanical changes after CXL.

An increase in activity of proteinase enzymes and reduction of proteinase inhibitors have been identified in keratoconic corneas^41^ and are liable to be important factors in the pathophysiology of the condition and disease progression. The main aim of corneal cross-linking is to halt the progression of corneal ectasia. While cross-linking the macromolecules within the corneal stroma undoubtedly augments its mechanical strength, it also increases its resistance to enzymatic digestion. Spoerl et al.^42^ were the first to demonstrate this increased resistance of the corneal stroma to enzymatic digestion after CXL. The ability of CXL to increase corneal stromal resistance to enzymatic digestion can therefore be expected to be a significant factor with regard to efficacy of the procedure in halting disease progression. To what extend and precisely how resistance to enzymatic digestion and increase in biomechanical strength after CXL are responsible for and contribute to its efficacy is yet undetermined, but both can undoubtedly be used as a measure of CXL efficacy. It is for such reasons, as well as poor measurement reliability of current methods of corneal biomechanical assessment (which we discussed above), that in this study we employed an enzymatic digestion methodology. We have previously demonstrated the efficacy of this methodology in determining differences in both ACXL and epithelium-on CXL protocols compared to SCXL.^17^,^23^

We employed two experimental runs in this study to ensure consistency and verify our dry weight measurements, as well as to simplify and improve our methodology. While daily measurements of the disc diameters until digestion do provide information about the structural integrity of the anterior layers of the cornea to which CXL is directed,^17^,^23^ their measurement, as well as being time-consuming, is subject to variation. This is especially true in the later stages of digestion, when the repeatability and hence reliability of the measurements are reduced due to the smaller dimensions of the tissue and its increasingly irregular outline. In addition, with time the pepsin solution in which the discs are immersed loses its digestive activity. Indeed, in the first experimental run we perceived that the discs had stopped digesting at about day 26; therefore, in order to complete the experiment, we had to renew the pepsin solution. As dry weight measurements, which represent the total mass of undigested tissue, were found to be highly reproducible because they negate any problems associated with within-sample variations in corneal thickness, shape, and between-sample differences in hydration, in the second experimental run we relied solely on this methodology and collected dry weight measurements at day 10 and day 20 of digestion.

All CXL-treated eyes in our study received an application of an iso-osmolar riboflavin solution containing 20% dextran. Consistent with previous studies,^17^,^23^,^43^ this resulted in a significant decrease in corneal thickness (Fig. 1), probably attributable to the deturgescent effect of the 20% dextran and dehydration of the de-epithelialized cornea during cross-linking. As might be expected, the concentration of riboflavin over the ranges we used had no effect on corneal thickness.

In previous studies using an identical methodology, we have shown that there are no differences at all between digestion times in nonirradiated corneal controls that have not received riboflavin drops and those soaked in riboflavin for 30 minutes.^16^,^23^ This allowed us, in this present study, to reduce the number of control groups and use only completely de-epithelialized, nontreated, nonirradiated corneas as a sole control group. As we have documented previously,^17^,^23^ the time required for complete digestion of the treated CXL corneal discs was significantly longer than the untreated controls, demonstrating the reproducibility of this technique in detecting changes between CXL-treated and untreated corneas. However, despite an evident trend for the higher-concentration riboflavin-treated corneas to take longer to digest completely, the differences between groups were not significant. In contrast, the dry weight measurements after 10 days of digestion (Figs. 3A, 3B; Table 3) and to a lesser extent at 20 days (Fig. 4; Table 4), demonstrated significant differences between the treatment groups, indicating that an increase in riboflavin concentration from 0.05% to 3% results in a progressive improvement in the resistance of cross-linked corneas to enzymatic digestion. This suggests that measurement of the time until digestion is complete is not sensitive enough to detect subtle (but probably clinically important) differences in CXL efficacy with differing protocols. Certainly, in a previous study using this methodology to investigate SCXL versus ACXL protocols,^18^ time to complete digestion showed no differences, whereas dry weight measurements indicated significant differences, with better results in SCXL.^17^ Clinical investigations confirmed and supported such laboratory findings of a reduced efficacy with ACXL.^18^,^19^

The similarity between runs 1 and 2 in terms of the average dry weight measurements for each group after 10 days of digestion confirmed our belief that the dry weight technique is a less time-consuming and a more accurate and reproducible technique for investigating CXL efficacy than are daily measurements of corneal diameter (Fig. 3A, 3B; Table 3).

Over the ranges we tested, such outcomes support a dose-response curve of riboflavin in CXL, with higher concentrations, up to 0.3%, achieving greater efficacy. How these results will transfer into clinical efficacy is undetermined until clinical trials with higher concentrations are undertaken, but they may have important implications. In this study, all treatments were conducted using an ACXL technique of 9 mW/cm^2^ for 10 minutes (total energy dose 5.4 J/cm^2^), yet manipulation of riboflavin dosage and hence stromal concentration resulted in improved efficacy. This occurred without oxygen supplementation or pulsing, which some authors, both in laboratory^23^ and clinical studies,^28^,^29^ have hypothesized may augment the CXL process, especially with ACXL protocols. A riboflavin dose-response curve might suggest that while oxygen and type II photochemical (aerobic) reactions are undoubtedly important in the CXL process,^24^ type I photochemical (anaerobic) pathways, with direct interaction between excited riboflavin triplets and stromal proteins resulting in cross-linking, play a significant role and may be augmented by increasing riboflavin concentrations during CXL. As such, the efficacy of ACXL might be improved not only by increasing the UVA dosage and perhaps the addition of supplemental oxygen and/or pulsing but also by simply increasing stromal riboflavin concentration. With manipulation of such parameters, it might be possible with ACXL to reduce the overall treatment time while maintaining the same clinical efficacy as SCXL.

Such results may also have important implications for epithelium-on CXL, where reports of reduced efficacy compared to SCXL^44^ are likely to be related to limited riboflavin absorption through the intact corneal epithelium and low stromal riboflavin concentrations. A dose-response curve of riboflavin with higher concentrations, at least up to 0.3%, achieving greater efficacy would support such a hypothesis. It is of note that by using two-photon fluorescence, we have previously shown that stromal riboflavin concentrations with currently commercially available protocols are only 10% to 30% of that achieved with SCXL^45^ and that in laboratory studies, manipulation of such epithelium-on protocols can result in higher achieved stromal riboflavin concentrations^46^ and resultant increased CXL efficacy.^47^

Finally, in the CXL process riboflavin has two basic functions. As well as acting as a photosensitizer to produce both oxygen singlets and riboflavin triplets to drive the CXL process,^22^ it also absorbs the UVA photons within the anterior corneal stromal to reduce UVA toxicity and potential damage to internal ocular structures such as the endothelium.^48^ The use of higher-strength riboflavin solutions resulting in increased stromal riboflavin concentrations and therefore increased UVA absorption within the anterior stroma, should theoretically reduce the amount of UVA radiation reaching deeper layers of the cornea, reducing the risk of endothelial toxicity as well as allowing for the possible treatment of thinner corneas.^49^ It is interesting that while it might be supposed that increased absorption of UVA photons within the anterior stroma by higher riboflavin concentrations might result in an increased but more superficial cross-linking effect, our dry weight results of a significant increase in the mass of residual undigested corneal tissue with increasing riboflavin concentration, while there were no differences in disc diameter measurements, suggest that this is probably not the case. We can postulate from such results that more volume of tissue is being cross-linked, including that in deeper stromal layers with higher riboflavin concentrations, and that we are not merely getting a more intense, but superficial effect, that one might expect not to affect the dry weight measurements but to result in differences in disc diameter measurements during the digestion process.

## Conclusions

Our results demonstrate a dose-response curve with increasing riboflavin solution concentrations up to 0.3% achieving greater CXL efficacy. This suggests that by simply increasing the riboflavin concentration, at least within the limits we tested, it may be possible to increase the efficacy of ACXL to match that of SCXL. The use of higher concentrations of riboflavin solution may also improve the outcomes of epithelium-on CXL as well and allow the safe treatment of thin corneas. Whether or not higher concentrations of riboflavin in CXL may result in any adverse complications (such as increased haze) is as yet undetermined, but this study appears to support the commencement of clinical trials of CXL with moderately increased higher riboflavin concentration solutions.
