# Erratum

**DOI:** 10.1167/iovs.17-22994a

**Published:** 2019-04

***Erratum in:*** “An Investigation of the Effects of Riboflavin Concentration on the Efficacy of Corneal Cross-Linking Using an Enzymatic Resistance Model in Porcine Corneas” by Naomi A. L. O'Brart, David P. S. O'Brart, Nada H. Aldahlawi, Sally Hayes, and Keith M. Meek (*Invest Ophthalmol Vis Sci*. 2018;59:1058–1065) https://doi.org/10.1167/iovs.17-22994

This article was originally published under the Creative Commons Attribution-NonCommercial-NoDerivatives (CC BY-NC-ND) license. It should have been published under the Creative Commons Attribution (CC BY) license. The license has been changed in the article online.

This work was funded by a Programme Grant MR/K000837/1 from the Medical Research Council (awarded to KMM). This information has been added to the Acknowledgments in the article online.

